# Warp-Knitted Spacer Fabrics: A Versatile Platform to Generate Fiber-Reinforced Hydrogels for 3D Tissue Engineering

**DOI:** 10.3390/ma13163518

**Published:** 2020-08-10

**Authors:** Benedikt Schäfer, Caroline Emonts, Nikola Glimpel, Tim Ruhl, Astrid S. Obrecht, Stefan Jockenhoevel, Thomas Gries, Justus P. Beier, Andreas Blaeser

**Affiliations:** 1 Department of Plastic Surgery, Hand Surgery-Burn Center, University Hospital RWTH Aachen, 52074 Aachen, Germany; bschaefer@ukaachen.de (B.S.); truhl@ukaachen.de (T.R.); aobrecht@ukaachen.de (A.S.O.); jbeier@ukaachen.de (J.P.B.); 2 Institut für Textiltechnik, RWTH Aachen University, 52062 Aachen, Germany; Caroline.Emonts@ita.rwth-aachen.de (C.E.); Nikola.Glimpel@ita.rwth-aachen.de (N.G.); Thomas.Gries@ita.rwth-aachen.de (T.G.); 3 Department of Biohybrid and Medical Textiles (BioTex), Applied Medical Engineering, Helmholtz Institute, RWTH Aachen University, 52074 Aachen, Germany; jockenhoevel@ame.rwth-aachen.de; 4 Institute for BioMedical Printing Technology, Technical University of Darmstadt, 64289 Darmstadt, Germany; 5 Centre for Synthetic Biology, Technical University of Darmstadt, 64289 Darmstadt, Germany

**Keywords:** tissue engineering, biofabrication, biohybrid scaffold, textile engineering

## Abstract

Mesenchymal stem cells (MSCs) possess huge potential for regenerative medicine. For tissue engineering approaches, scaffolds and hydrogels are routinely used as extracellular matrix (ECM) carriers. The present study investigated the feasibility of using textile-reinforced hydrogels with adjustable porosity and elasticity as a versatile platform for soft tissue engineering. A warp-knitted poly (ethylene terephthalate) (PET) scaffold was developed and characterized with respect to morphology, porosity, and mechanics. The textile carrier was infiltrated with hydrogels and cells resulting in a fiber-reinforced matrix with adjustable biological as well as mechanical cues. Finally, the potential of this platform technology for regenerative medicine was tested on the example of fat tissue engineering. MSCs were seeded on the construct and exposed to adipogenic differentiation medium. Cell invasion was detected by two-photon microscopy, proliferation was measured by the PrestoBlue assay. Successful adipogenesis was demonstrated using Oil Red O staining as well as measurement of secreted adipokines. In conclusion, the given microenvironment featured optimal mechanical as well as biological properties for proliferation and differentiation of MSCs. Besides fat tissue, the textile-reinforced hydrogel system with adjustable mechanics could be a promising platform for future fabrication of versatile soft tissues, such as cartilage, tendon, or muscle.

## 1. Introduction

Mesenchymal stem cells (MSCs) are multipotent progenitor cells in the stroma of several tissues in the body, which can differentiate in several cell types of the mesodermal lineage, i.e., adipocytes, osteoblasts, chondrocytes, and myocytes [1]. They have been defined by the Committee of the International Society for Cellular Therapy by their ability to differentiate and by being plastic adherent in standard culture conditions [2]. MSCs can be obtained from various tissues, especially bone marrow and adipose, and are used as cell sources for therapeutic or experimental exploitation [3]. Adipose tissue is a composition of regenerative cell types containing not only adipocytes, but also MSCs, including adipose-derived stromal cells, and preadipocytes [4,5]. It is nowadays one of the most relevant cell sources, because adipose tissue contains approximately 500 times more MSCs compared to bone marrow [5]. In addition, adipose tissue can be harvested more easily and less invasive than bone marrow, e.g., by liposuction [4].

For the above-mentioned reasons, adipose tissue-derived stromal cells are of great interest in the field of regenerative medicine and tissue engineering and offer an ideal cell source for the treatment of large size soft tissue defects. The current gold standard for treating such defects, e.g., breast reconstruction after mastectomy or defects after trauma, is the transplantation of autologous tissue, which is always associated with a second operation field and the risk of donor site morbidity [6,7,8]. Thus, several approaches target at the generation and transplantation of artificial tissue to repair damaged or replace lost tissue. As a growth matrix, biomaterial-derived scaffolds are widely used in tissue engineering, in both in vitro and in vivo experiments [9,10]. Besides rigid metal, polymer, or ceramic scaffolds, hydrogels gained special attraction as matrix material for soft tissue regeneration [11,12,13]. They provide a good environment for three-dimensional cell culture due to their high water content, their ECM-like structure, and the presence of cell adhesion motifs [14,15]. Numerous studies have described the biochemical and biophysical properties of hydrogels and their excellent ability to mimic the cell physiological microenvironment [16]. In particular, the effect of spatiotemporal modulated mechanics on stem cell fate is in the focus of attention [17]. However, hydrogels lack mechanical strength and stability. For this reason, their clinical applicability is very limited. Mechanical strength and stiffness are not only of importance in load-bearing implants, such as bone or cartilage, but also play a major role in soft tissue mechanics. For instance, to enable safe scaffold handling and suture retention during surgery, even hydrogel–cell constructs for soft tissue repair must withstand mechanical stress. To overcome the shortcoming of poor mechanical properties, while maintaining their elastic nature, hydrogels can be reinforced with textile structures. The basic stability of the adipose tissue is given by the superficial fascia system contained therein. This fascia layer ensures stability and displacement of the soft tissue. It also provides skin attachment to underlying structures. Thus, stability through body fascia in the apparently unstable adipose tissue is highly relevant.

**Scheme 1:**
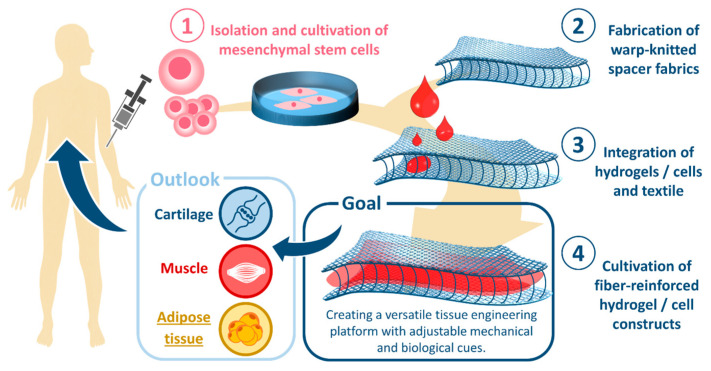
Schematic illustration of the concept, experimental procedure, goal, and outlook of the study. Using mesenchymal stem cells, hydrogels, and warp-knitted spacer fabrics, a versatile tissue engineering platform with adjustable mechanical as well as biological cues is developed. The potential of the described approach is tested on the example of adipose tissue. Besides adipose tissue, the platform holds great promise for versatile soft tissue engineering applications, including muscle, cartilage, or tendon, in the future.

Up to now, successful textile reinforcement could be shown for thin, planar, and cylindrical structures, such as heart valves or blood vessels [18,19], using 2D textile meshes. The goal of the current study is to investigate the potential of 3D textile morphologies as building block for the generation of thick soft tissue structures. In particular, the mechanical and biological properties of warp-knitted spacer fabrics and their applicability as adipose cell matrix will be elucidated in the present study (Scheme 1). Due to their well-adjustable porosity, pore size, and Young’s modulus, we consider spacer fabrics an extremely versatile scaffold not limited to fat tissue applications but also suitable for a broad range of soft and hard tissues, such as bone, cartilage, and muscle.

## 2. Material and Methods

### 2.1. Spacer Fabric Development and Fabrication

The spacer fabrics contained two different poly (ethylene terephthalate) (PET) fibers. The multifilament used for both cover areas comprised 24 fibers with a total fineness of 78 dtex. Due to the higher bending stiffness, a PET monofilament with a fineness of 45 dtex was used for the pile yarn. For both cover areas as well as the pile yarn geometry of the scaffold different warp-knitting settings were applied (Table 1). All lappings were produced using a double-bar raschel warp-knitting machine (DR 16 EEC/EAC, KARL MEYER Holding GmbH &Co. KG, Obertshausen, Germany).

### 2.2. Laser Cutting

To cut the produced textile samples in a cell culture, usable format circular samples were cut out using laser cutting technology (Magflex OR laser, O.R. Lasertechnologie GmbH, Dieburg, Germany). To reduce heat-related damages, the spacer fabric was placed on an aluminum plate for optimal heat conduction during cutting. The adjusted parameters included the mark speed (5000), power (90%), modulation frequency (100), and repeat count (350).

### 2.3. Spacer Fabric Morphology

The most important morphological scaffold characteristics, such as porosity, pore size, and thickness, were evaluated using microcomputer tomography (CT-Alpha, Procoon X-Ray GmbH, Sarstedt, Germany). By means of the μ-CT a multitude of cross-sectional images was produced and reconstructed to a three-dimensional model. Three samples were taken from different parts of the fabric. The adjusted parameters were X-ray tube voltage: 35 kV, X-ray tube current: 200 mA, exposure time: 150 ms, detector position: 300, sample position: 595, and sample size (L × W × D): 2.5 cm × 1.2 cm × 0.15 cm.

### 2.4. Pore Size, Porosity, and Dimensions of the Spacer Fabric

To evaluate the pore size and porosity from the three-dimensional µ-CT model of the spacer fabric, the software module PoroDict in the program GeoDict (GeoDict, Software module: PoroDict, Math2Market GmbH, Kaiserslautern, Germany) was used. After loading and adjusting the model to a coordinate system, the sample volume could be adjusted such that no surplus cavities were included in the measurement. The classification of fiber and cavity was done by assignment of the gray values. The pore size was determined by simulating a sphere in the center of each cavity, which grew incrementally until touching the fibers. The results were given in percentage of pores within a specific range (200 to 500 μm). Furthermore, the amount of cells, which were located above and below the pores were analyzed.

The porosity Φ of a spacer fabric is defined as the ratio between the cavity volume (V_c_) and the total volume (V_total_): Φ = V_c_/V_total_. The results of the porosity measurements were given as mean value of the three samples. To determine the pore size distribution of the cover areas and to measure the thickness of the spacer fabric the software VGStudioMax (Volume Graphics GmbH, Heidelberg, Germany) was applied. The inner and outer sizes were measured at three randomly chosen sites in three different slices of each sample.

### 2.5. Hydrogel Preparation

In this study, hydrogels based on agarose, collagen, and a blend of agarose and collagen were applied. The agarose gel was prepared by mixing the desires amount of Millipore water with agarose powder (Agarose, low gelling temperature, Sigma-Aldrich Cooperation, St. Louis, MO, USA). The mixture was placed in the autoclave at 121 °C for 15 min to dissolve the agarose powder. For all experiments a concentration of 0.5% (wt/vol) were used. The collagen gel (Collagen G, L7213, Biochrom GmbH, Berlin, Germany) was processed according to the protocol of Biochrom. First, sterile 0.7 M NaOH (A 3910, AppliChem GmbH, Biochemica, Darmstadt) and 1 M HEPES (Hepes Sodium Salt, H 3784, Sigma Lifescience, St. Louis, USA) buffer were prepared. These two solutions were mixed in equal parts. The resulting solution, solution A, was mixed in equal parts with 10x medium. For the current application, 10x DMEM (L083637, Biochrom GmbH, Berlin, Germany) was used. The resulting mixture, solution B, was mixed in the ratio 1:4 with the 0.4% collagen G solution. Finally, the agarose–collagen blend was prepared by mixing both substances in a 1:1 ratio.

For the preparation of textile-reinforced hydrogel composites, the hydrogels (collagen, agarose–collagen, or agarose) were filled in the spacer fabric or casted in the cell-crown mold in a well plate. The spacer fabrics were filled first with a small amount of gel (10 μL for 96-well plates; 100 μL for 24-well plates) to seal the lower cover area. After gelation of this part, the rest of the gel (40 μL for 96-well plates, 300 μL for 24-well plates) was added.

### 2.6. Rheological Characterization of the Hydrogel

The rheology of hydrogels is an important property to determine their flow behavior and their suitability for textile scaffold infiltration. In this context, the hydrogel viscosity is of particular interest. The viscosity of the prepared agarose hydrogels were measured using a rotational rheometer (Kinexus ultra+, Malvern, UK) and the software “rspace for Kinexus”. A shear ramp test (0.1 1/s to 10^4^ 1/s) was carried out with a ramp time of 30 min. In addition, the gelation point of the agarose hydrogel was measured using a temperature ramp test. The loss (G″) and storage (G′) modulus were recorded during cooling to describe the visco-elastic response of the hydrogel. The start and end temperatures were set to 37 °C and 15 °C, respectively, while the test was conducted with a ramp rate of 5 °C/min. Shear strain was set to 1%. For both experiments, three samples with a volume of 290 µL were tested.

### 2.7. Mechanical Characterization of Hydrogel-Laden Spacer Fabrics

All samples were stored under standardized atmospheric conditions for at least 12 h before testing.

#### 2.7.1. Uniaxial Tensile Test

To determine the maximum force and the elongation at maximum force as well as the Young’s modulus under tensile stress, a uniaxial tensile test using the strip method was conducted. The measurements were carried out using a Zwick Roell 1455 testing machine (Zwick Roell 1455, Zwick Roell AG, Ulm, Germany) with a load cell of 5 kN X-Force HP. Clamps with a corrugated polymer surface made of vulkollan were used. The applied preload was 5 N, the clamping length was 200 mm, and the sample width was 25 mm. To calculate the Young’s modulus the measured forces were converted into stresses by referring to the thickness of the sample. In this step, the cross section of the textile was assumed to be a bulk material. For each spacer fabric design, five individual samples were tested. The testing was conducted according to DIN EN ISO 13934.

#### 2.7.2. Unconfined Compression Testing

To evaluate the reinforcing effect of the spacer fabric regarding compressive loads, an unconfined compression test was carried out. In this experiment the textile scaffold, a hydrogel, as well as a textile-reinforced hydrogel (composite of hydrogel and spacer fabric), were tested and compared with each other. In preparation for the measurement, all tested samples were placed in 6-well plates. The hydrogel samples were prepared by molding hydrogel in cell crowns. After gelation the bottom membrane was peeled off and the hydrogel was placed in the well plate. The samples measured 26 mm in diameter and 3 mm in height. The textile samples were cut by a laser cutter in equal dimensions. To prepare the composite scaffolds (textile and hydrogel) the laser cut textile samples were subsequently filled with 500 µL of the respective hydrogel. All tests were conducted using the universal mechanical testing machine Zwick Z2.5 (Zwick Z2.5, Zwick Roell AG, Ulm, Germany) with a load cell of 5 N (hydrogel samples) or 10 N (textile and composite samples). The following testing parameters were applied; pre-load for textile and composite samples: 1 cN, stamp size: 875 mm^2^, testing velocity: 1 mm/min, distance at starting position: 0.3 mm.

#### 2.7.3. Biaxial Tensile Testing

Biaxial tensile testing is a new testing method to determine the biaxial mechanical properties of materials. Here, this method was used to study the mechanical tensile properties of warp-knitted textile scaffolds. The tests were carried out with the biaxial testing machine of Zwick (Biaxial Testing Machine, Zwick Roell AG, Ulm, Germany). The biaxial testing device comprises four movable traverses. For contactless elongation measurements a video and laser extensometer are installed. All samples were tested in water at 37 °C. To ensure defined start conditions, a preload of 20 mN was applied. All four traverses were moved with a uniform velocity of 3 mm/min. If the lengthwise distance change was higher than the crosswise distance change, the traverses in length direction were stopped until the traverses in crosswise direction had reached the according distance, and vice versa. This procedure ensured a synchronous elongation of the samples in length and cross direction. Thus, uniform elongation ratios in both directions could be obtained despite the anisotropic properties of warp-knitted spacer fabrics. The iterative process was conducted until 1 mm elongation was reached, which corresponded to 10% elongation of the sample. Afterwards, the samples were relaxed by moving the traverses back to their starting position with 3 mm/min. The testing procedure contained five repetitive cycles. During testing the readings were recorded every 0.02 s.

### 2.8. Cell Culture Materials

PrestoBlue, fetal bovine serum (FBS), high glucose medium (4.5 g/L), and Dulbecco’s Modified Eagle’s medium (DMEM/F-12) were obtained from Life Technologies (Life Technologies, Carlsbad, CA, USA). Collagenase (type I) was purchased from Worthington Biochemical Corp. (Worthington Biochemical Corp., Lakewood, CA, USA). Paraformaldehyde (PFA), trypsin–EDTA, penicillin–streptomycin, and Tween^®^20 were obtained from Sigma (Sigma Aldrich, St. Louis, MO, USA). Acetic acid and crystal violet were bought from Roth (Carl Roth GmbH, Karlsruhe, Germany). Basic fibroblast growth factor (bFGF) was obtained from PeproTech (PeproTech GmbH, Hamburg, Germany). Isopropyl alcohol Oil Red O from Merck (Merck KGaA, Darmstadt, Germany) was used. phosphate buffered saline (PBS) was bought from Biochrom (Biochrom GmbH, Berlin, Germany). Rosiglitazone was from LKT Laboratories Inc. (LKT Laboratories Inc., St Paul, MN, USA). The study protocol was approved by the regional ethics committee (Ethics Committee of the RWTH Aachen University Faculty of Medicine, Aachen, Germany; EK163/07).

### 2.9. Cell Isolation and Culture Procedure

Subcutaneous adipose tissue was taken from abdominoplasty of two healthy patients. The donors had been informed about the use of the cells from their tissue and had given informed consent. The study protocol was approved by the regional ethics committee (Ethics Committee of the RWTH Aachen University Faculty of Medicine, Aachen, Germany; EK163/07). Isolation of adipose tissue MSCs and their cultivation followed the procedure described elsewhere [20]. Briefly, adipose tissue was digested in 0.2% collagenase solution for 45 min at 37 °C. The mature adipocyte fraction was separated from the stromal cell fraction by centrifugation (400 *g* for 10 min at RT). Subsequently, the stromal fraction was filtered through a 250 nm nylon mesh (neoLab, Heidelberg, Germany) and centrifuged afterwards. The cells were resuspended in PBS and centrifuged again. The cells were cultured in proliferation medium (DMEM with 0.1% bFGF, 10% FBS, Pen/Strep at 100 U/mL). Adipogenic differentiation was induced by differentiation medium (Diff), consisting of high glucose medium supplemented with 10% FBS, insulin (10 µg/mL) and rosiglitazone (0.2 µg/100 mL). The medium was replaced every 2–3 days for 12 days.

### 2.10. MSC Seeding on Spacer Fabric Scaffold

From the primary cultures, experiments were conducted with cells from passage 4. Therefore, MSCs were trypsinized, then they were washed and resuspended in proliferation medium. From this cellular stock solution (10^6^ cells per mL), respective volumes were pipetted on UV-sterilized PET scaffolds (diameter: 5 mm, height: 1 mm) for seeding them with 50, 100, 200, and 400 × 10^3^ cells. A 2D control was performed with 100,000 cells on a 12-well plate for proliferation and differentiation experiments. The knitted fabrics (n = 10 per cell number) were then placed on the bottom of 12-well plates and covered with 2 mL of either proliferation or differentiation medium. Successful adherence has been approved during control trials by two-photon laser scanning microscopy after fluorescein diacetate staining (FDA-stock, 5 mg/1 mL acetone) 2 days after seeding. After removal of the proliferation medium, the scaffolds were washed with PBS and incubated with FDA staining solution (5 mL proliferation medium without FBS supplemented with 8 µL FDA stock) for 5 min at RT in the dark. Scaffolds were washed with PBS and analyzed under a microscope (FV1000MPE, Olympus, Tokyo, Japan).

### 2.11. Cell Culture Assays

#### 2.11.1. Presto Blue Assay

Two days after seeding the cells on the PET-scaffolds, data acquisition began (Day 0). Therefore, the cell-loaded scaffold was carefully lifted with sterile forceps and transferred into a new well (12-well plate), with freshly prepared PrestoBlue (PB) containing medium (200 µL PB in 2 mL proliferation medium). The remaining cell-conditioned medium was collected and frozen for ELISA measurements. The PrestoBlue (PB) assay followed the manufacturer’s instructions. After 1 h of incubation, 100 µL of the medium was transferred into a 96-well plate and fluorescence was measured in triplets in a microplate reader (BMG Labtech, Ortenberg, Germany) at wavelength of 590 nm. Following the PB measurements, the scaffold was again transferred to a new well (12-well plate) and prepared with 2 mL proliferation medium. Metabolic activity was repeatedly measured for a period of 28 days (Day 7, 14, 21, and 28).

#### 2.11.2. Crystal Violet Assay

Crystal violet staining followed the protocol of Gillies et al. with slight modifications [1]. Briefly, cells (in 2D and 3D experiments) were washed with PBS and fixed in iso-propanol (10 min at RT). After washing with 0.05% Tween 20 in PBS, cells were stained with a 0.1% crystal violet solution (20 min at RT). The cells were washed afterwards with distilled water. Adsorbed crystal violet was diluted in 300 µL acetic acid (33%) per well. Following an incubation period of 15 min, 70 µL per sample were transferred in triplets to an optical plate and absorbance was quantified at 620 nm.

#### 2.11.3. Oil Red O-Staining

After 12 days of adipogenic differentiation, Oil Red O staining was performed as described earlier [20]. The cell supernatants were collected for ELISA measurements of adipokines. Adipogenesis-induced preadipocytes/adipocytes in wells (2-D) and on scaffolds (3-D) were washed with PBS and fixed for 20 min in 4% PFA solution in PBS. Fixed cells were washed in PBS and stained at room temperature with Oil Red O in 60% isopropanol. After 15 min, cells were gently washed in distilled water. Images of stained cells were taken up under a light microscope (EVOS Auto FL 2, ThermoFisher Scientific, Darmstadt, Germany).

#### 2.11.4. Enzyme-Linked Immunosorbent Assay (ELISA)

To measure the content of the adipokines leptin and serpin, the supernatants of differentiating cells in wells (adipogenic differentiation in 2D) and on scaffolds (adipogenic differentiation and proliferation in 3D) were analyzed using the respective Leptin and Serpin Enzyme-Linked Immunosorbent Assay Duo-Sets (R&D Systems, Minneapolis, MN, USA). Extinction was measured as recommended by the manufacturer using a microplate reader at 450 nm using the reference value of 540 nm.

### 2.12. Statistics

All data of experiments with MSCs were expressed as means and standard error of the mean (±). Data of PrestoBlue experiments were grouped in order to evaluate the metabolic activity for each day of testing and analyzed by two-way ANOVA (analysis of variance) with repeated measures, followed by the Bonferroni post hoc test for pairwise comparison between factors. Crystal violet and ELISA data were tested for significance with the unpaired Student’s *t*-test (SPSS 25, SPSS Inc., Chicago, IL, USA). Statistical significance was accepted if *p* < 0.05.

## 3. Results

### 3.1. Biofabrication of Textile-Reinforced Hydrogels Scaffolds

The present study describes a novel approach to generate fiber-reinforced hydrogel scaffolds for tissue engineering applications. PET spacer fabrics were produced using warp-knitting technology. These scaffolds were further combined with polysaccharide- and protein-based hydrogels as well as living cells. The morphology and mechanics of these hybrid scaffolds were studied together with their cell biological response.

**Figure 1:**
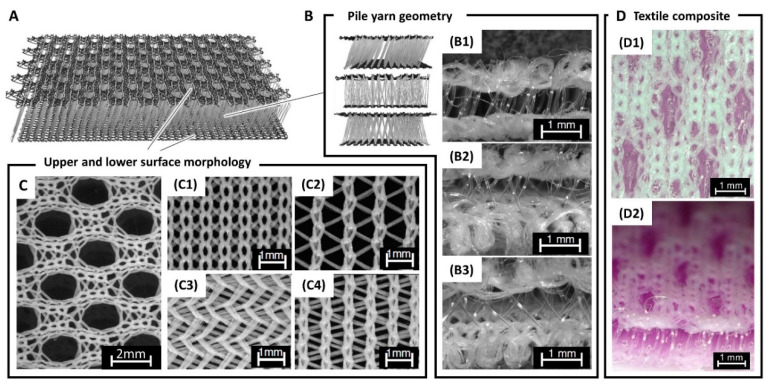
Warp-knitted spacer fabric design. The fabricated spacer fabrics comprised an upper and lower surface/cover area as well as several pile yarns (**A**). The pile yarn connects the upper and lower cover area and could be arranged in three different geometries (**B1**–**B3**). For the majority of the experiments conducted in this study the geometry displayed in B2 was applied. In addition to the pile yarn geometry the morphology of the cover areas was varied (**C**, **C1**–**C4**). Finally, the warp-knitted spacer fabrics were infiltrated with (cell-laden) hydrogel to generate fiber-reinforced composites. Pictures of exemplarily fabricated composites are illustrated in top (**D1**) and side view (**D2**).

Spacer fabrics are three-dimensional textile scaffolds that comprise an upper and lower cover area connected with multiple pile yarns (Figure 1A). Depending on the machine settings different pile yarn geometries can be applied (Figure 1B). For the cover areas, a broad range of designs and morphologies can be selected from (Figure 1C).

**Figure 2:**
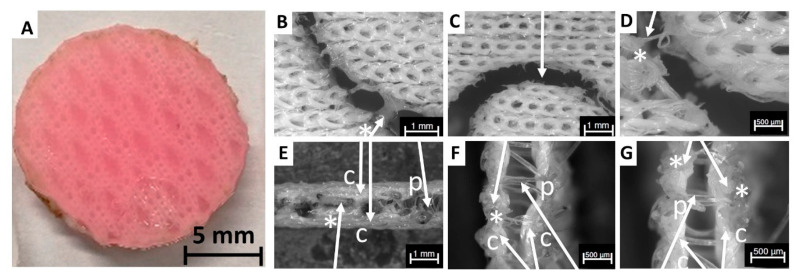
Photography of the laser-cut circular spacer fabric sample loaded with hydrogel (**A**). The laser cutting process was associated with a couple of challenges regarding proper separation of the sample from the textile sheet (**B**–**G**). The laser induced heat resulted in partially melted poly (ethylene terephthalate) (PET) fibers (indicated with an asterisk *). These could either impede separation of the sample (**B** and **D**) or lead to partial occlusion of the lateral pores (**E**–**G**). The cover areas (“C”) and pile yarns (“p”) are also indicated with arrows. Under ideal cutting conditions and using a metal block below the sample to increase heat conduction during cutting a clear cut of the sample could be obtained (**C**).

Following the fabrication process, the spacer fabrics were cut into circular samples with diameters 5, 15, and 26 mm using a laser cutter (Figure 2). During laser cutting, different effects concerning the cutting quality could be observed (Figure 2B–G). Due to heat generation, parts of the cover areas and pile yarns melted hampering the separation of the samples. In addition, the melted material could result in partially occlusion of the open porous scaffolds geometry. Under optimal conditions clearly cut samples with an open, non-glued pile yarn geometry could be produced (Figure 2C,G).

**Figure 3:**
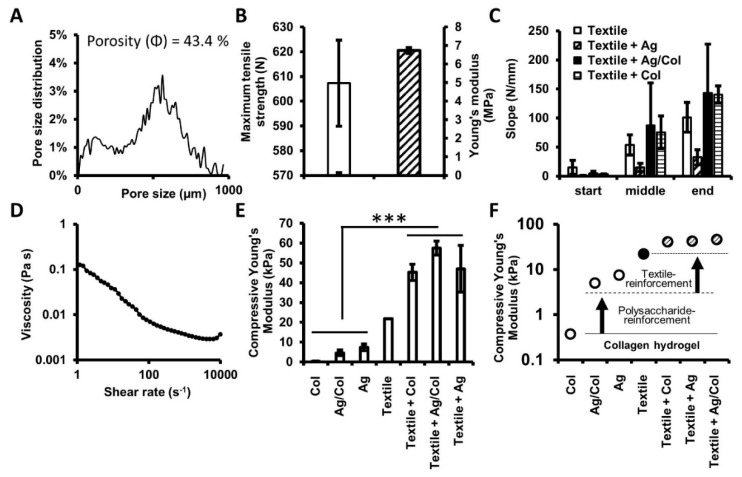
Morphological and mechanical analysis of warp-knitted spacer fabrics. The porosity and pore size distribution of the scaffold was calculated by analyzing a µ-CT scan of the textile (**A**). In an uniaxial tensile test, the maximum tensile strength as well as the Young’s modulus were determined (**B**). In a biaxial tensile test the performance of the textile was tested and compared with textile reinforced hydrogels (**C**). In order to determine the flow behavior of the applied gels the viscosity was measured (**D**, agarose–collagen blend) and the power law constants (k and n) were calculated. Finally, the compressive Young’s moduli of non-reinforced hydrogels, the textile carrier, and textile reinforced hydrogels were assessed (**E**). Significance levels were *p* < 0.05 (***). The results indicate that the mechanical behavior of collagen hydrogels can be tuned over several orders of magnitude (**F**), first by polysaccharide blending (from 10^0^ to 10^1^ kPa), and second by textile reinforcement (from 10^1^ to 10^2^ kPa).

The morphology, in particular the thickness, pore size, and porosity, of the fabricated scaffolds was analyzed using µ-CT imaging. The warp-knitted spacer fabrics had a thickness of 1.56 ± 0.06 mm, with a pile yarn height of 1.03 ± 0.09 mm. Porosity and pore size are important features for a tissue engineering scaffold as they might impact cell attachment, cell proliferation, as well as nutrient diffusion and removal of metabolic substances. The porosity (Φ) of the produced spacer fabrics was measured to be 43.4% ± 1.45% (Figure 3A). The pore size distribution peaked at ~560 µm and had an additional local maximum at ~120 µm. In more detail, 12.5% of the pores were below 200 µm, the majority (51.4%) were in the range of 200 to 500 µm, and 36.1% were bigger than 500 µm.

Next, the spacer fabric’s mechanical properties were assessed. The spacer fabric’s tensile properties were tested in a uniaxial as well as a biaxial experimental set-up. In the uniaxial tensile test, the scaffolds withstood an averaged maximum tensile force of 607.284 ± 19.52 N before break and exhibited a respective tensile Young’s modulus of 6.74 ± 0.17 MPa (Figure 3B). In the biaxial testing, the bare textile scaffold was measured and compared with hydrogel filled composites. In contrast to the conventional uniaxial testing, the results are given as the measured slope of the force/strain curve (Figure 3C). For each measurement, the samples were tested in 5 iterative stress/strain and relaxation cycles. During the repetitive measurement, it could be observed that the slope of the curve increased with every cycle (start, middle, end of the cycles), which can be contributed to a variation in the textile’s structural elasticity. The same effect could be detected for the hydrogel filled spacer fabrics (Figure 2C). Interestingly, the agarose filled textile exhibited a significantly lower slope compared to the bare textile and the textile-reinforced agarose–collagen and collagen sample.

To determine the rheological behavior of the applied hydrogels a viscosity measurement was conducted (Figure 3D). For agarose, the zero shear viscosity was determined to be 30 ± 2.6 mPa.s. The first Newtonian plateau was reached at a shear rate of 1.76 ± 0.40 s^−1^. The zero shear viscosity of the agarose–collagen blend was measured to be 86 ± 2.4 mPa.s. For agarose–collagen, the first Newtonian plateau was detected at a shear rate of 1.74 ± 0.18 s^−1^. The rheological measurements of collagen showed no clear first Newtonian plateau. Furthermore, the flow consistency index k and the flow behavior index n, applied in power-law modeling of the fluids, were calculated. The measurements resulted in flow consistency indices of 37 ± 4 mPa.s for agarose, 105 ± 2.5 mPa.s for agarose/collagen, and 69 ± 2.5 mPa.s for collagen. The flow behavior index was measured to be 0.985 ± 0.008 for agarose, 0.907 ± 0.0059 for agarose/collagen, and 1.03 ± 0.039 for collagen.

Finally, the compressive Young’s modulus of the applied hydrogels, the textile scaffolds, and the textile reinforced hydrogels was measured (Figure 3E). The non-reinforced hydrogels exhibited rather low Young’s moduli ranging from 0.4 kPa (collagen) to 4.5 kPa (agarose–collagen) and 7.4 kPa (agarose). The textile scaffold showed a significantly increased compressive stiffness (21.8 kPa). Interestingly, the three textile reinforced hydrogels exhibited the highest moduli ranging from 45.3 to 57.6 kPa.

### 3.2. Cellular Viability

**Figure 4:**
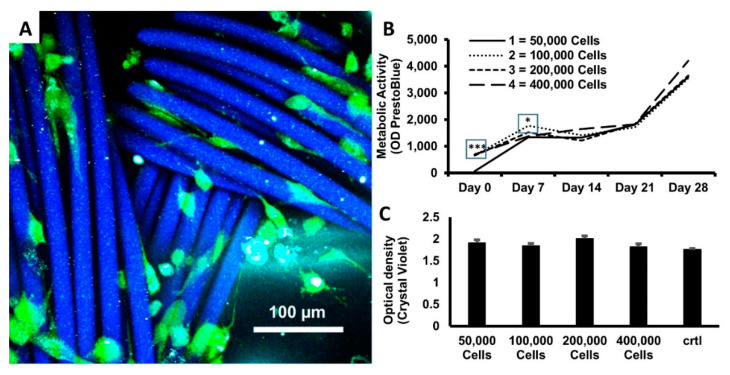
Two-photon microscopy of mesenchymal stem cells (MSCs) seeded on the fiber scaffold (**A**). The cells were stained with fluorescein diacetate (green) after two days of 3D culture. Repetitive measurements of metabolic activity using PrestoBlue conversion (mean values of the optical density) was performed over a period of 28 days (**B**). Cells were seeded at different densities (colored graphs) and the final cell numbers were quantified by Crystal violet staining at the end of the experiment (**C**). MSCs in 2D served as control (ctrl) for reference. Bars represent mean values + SEM. The number of experiments were as follows; for panels (b,c) n = 10 for each day of testing, except for ctrl, n = 3. Statistical analysis was performed by two-way ANOVA followed by pairwise comparison with the Bonferroni post hoc test; * *p* < 0.05, *** *p* < 0.001.

Cellular viability was assessed by the Presto Blue (PB) assay for investigation of the cells’ metabolic activity, as well as by crystal violet staining for evaluation of the final cell amount at the end of the experiment. The vitality of MSCs was measured by the PB assay and served as indicator for cellular proliferation over the course of the complete experiment [20]. After seeding the cells onto the knitted fabric, the cells attached to the fiber surface and were found equally distributed inside the scaffold (Figure 4A). The initial PB conversion of 100,000–400,000 cells was all on similar levels. This suggests that in these approaches the same amount of cells adhered to the scaffold. Seeding 50,000 cells in a smaller volume of cell suspension reduced the PB data at Day 0 (Figure 4B). On the following days of measurements, the metabolic activity of all cell numbers increased in a comparable manner reaching similar levels beginning with Day 14, followed by a parallel development until the end of the experiment. Two-way ANOVA with repeated measures after Greenhouse–Geisser correction determined significant differences between the days (F_days_(2.544, 91.587) = 461.38, *p* < 0.001), between the cell numbers (F_cell numbers_(3, 36) = 5.889, *p* = 0.002), and the interaction between both factors (F_days × cell numbers_(7.632, 91.578) = 3.118, *p* = 0.004). The following Bonferroni post hoc test found significant differences in metabolic activity on Day 0 between 50,000 cells and all other groups (*p* < 0.001), as well as between 50,000 and 100,000 cells at Day 7 (*p* = 0.025) (Figure 4B). After the final PB measurement on Day 28, the relative cell number was assessed by crystal violet staining in order to approve the PB data. Cell numbers were all on similar levels after 28 days of regular culture (Figure 4C). One-way ANOVA did not detect significant differences between the groups (F(3, 36) = 1.677, *p* = 0.189). For comparison, a 28-day 2-D culture of initially 100,000 MSC (n = 3) on a 12-well plate (surface area ~3.8 cm^2^) was stained with crystal violet, also (Figure 4C), which resulted in a staining level equal to the scaffold culture experiments. This suggests a comparable cell proliferation at both culture conditions.

### 3.3. Cell Differentiation

**Figure 5:**
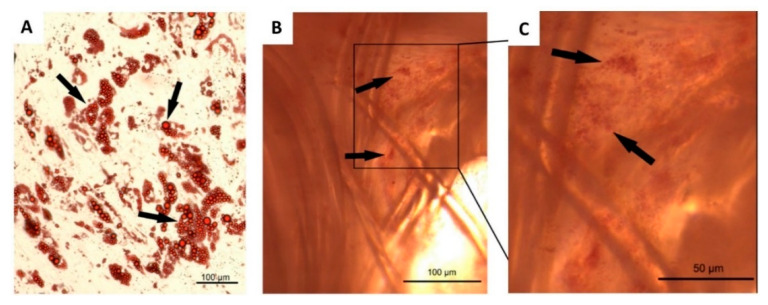
Oil Red O staining of preadipocytes during adipogenesis. Cells were cultured in 2-D (**A**) or in a 3-D scaffold (**B**). To improve visualization cells cultured in 3-D scaffolds were imaged with higher magnification (**C**). The arrows point out labeled lipid droplets of developing adipocytes.

**Figure 6:**
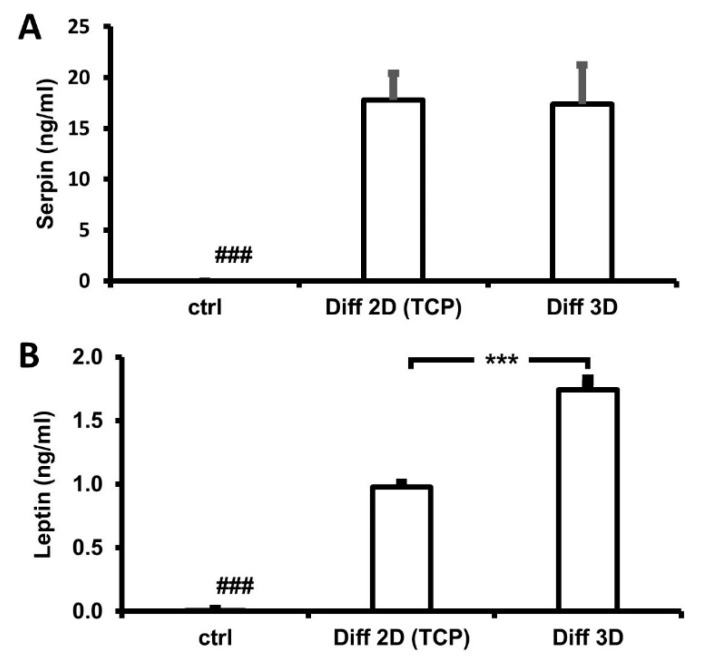
Influence of culture conditions on adipokine release of preadipocytes. Cells were left unstimulated (ctrl) or were exposed to adipogenic differentiation medium (Diff), either in 2-D or on a scaffold in 3-D. After 10 days of treatment, media were collected to determine protein secretion by sandwich ELISA (serpin, leptin). Data are expressed as mean values + SEM. The number of experiments were as follows; serpin (**A**), n = 24; leptin (**B**), n = 10. Statistical analysis was performed by the Student’s *t*-test for unpaired data; ### *p* < 0.001 vs. ctrl, *** *p* < 0.001 between 2-D and 3-D.

Adipogenic differentiation was induced by culturing MSCs for 10 days in differentiation medium and assessed using Oil Red O staining as an indicator of intracellular lipid accumulation [21]. Lipid droplets were clearly stained in 2D culture (Figure 5A), whereas staining in the 3D cultures was hard to identify due to thickness and consistency of the fiber network. However, we found labeled cells also on the scaffold (Figure 5B). For further detection of successful adipogenic differentiation, we measured the levels of two adipokines, which were exclusively released from adipocytes during development and maturity. Cell supernatants from untreated cells as well as 2D and 3D cultures exposed to differentiation medium were analyzed for their content of leptin and serpin. The latter was below detection level in the untreated control, while the basal leptin was at 2 pg/mL (Figure 6B). Adipogenic differentiation of MSCs significantly increased the release of both cytokines. For serpin, the measures were on comparable levels (1.7 ng/mL), while in 3D culture differentiation of the leptin levels was significantly increased compared to the 2D condition (Figure 6A).

## 4. Discussion

In this study, a novel approach for the biofabrication of fiber-reinforced hydrogels with adjustable morphological characteristics and mechanics is presented. Three-dimensional PET spacer fabrics were generated using warp-knitting technology. The fabrics were characterized with respect to their morphological as well as mechanical features. The results of this study outline the strength and versatility of spacer fabrics for tissue engineering applications. The properties of the scaffold can be adjusted at multiple levels: the fiber type and material, the design and binding of the cover areas, and the pile yarn geometry (Figure 1). Cutting the fabricated textiles into individual samples without influencing the material properties and whilst maintaining its lateral porosity was shown to be a particular challenge (Figure 2). However, with specific cooling and heat conduction circular samples ranging from 5 to 26 mm could be prepared. The fabricated samples exhibited an overall porosity of 43.4% and a broad pore size distribution peaking at a size of 560 µm (Figure 3A). The porosity is high enough to enable reproducible hydrogel infiltration and to provide sufficient nutrient supply. Finally, the mechanical characteristics of the hydrogels, the spacer fabric, and the composites were investigated. A collagen biofunctionalized polysaccharide-based hydrogel was applied. The cell biological, microstructural, and rheological properties of the agarose–collagen material system were intensively studied in previous work [22,23,24,25,26,27,28]. The material provides excellent stem cell proliferation as well as differentiation capacity. In addition, the blend’s angiogenic potential was previously described [28,29]. The current study focused on enhancing the bulk mechanical and shape fidelity properties of this highly biofunctional hydrogel system. In this context, the presented results emphasize the advantage of the biohybrid, textile-integrating approach. Fiber-reinforced hydrogel composites exhibited a significantly enhanced compressive modulus compared to their native counter parts (Figure 3E). Interestingly, the results further indicate that by combining polysaccharide blending and spacer fabric reinforcement, the mechanics of collagen hydrogels, which are still considered the gold-standard in tissue engineering, can be modulated over two orders of magnitude (Figure 3F). Besides the integration of textiles and fibers, in the literature other methods for the mechanical reinforcement of hydrogels are described, such as blending and dual crosslinking of natural and synthetic materials [30], or hybrid bioprinting, where hydrogels are deposited in parallel with biodegradable polymers [29]. While blending and dual crosslinking allows the targeted adjustment of the mechanical properties of hydrogels in the lower elasticity range (e.g., 5–25 kPa) [30], comparatively stiff scaffolds can be achieved by hybrid bioprinting (e.g., 30–50 MPa) [31]. In this context, the presented strategy offers certain advantages with respect to its broad adjustability. Using the described fiber-reinforced hydrogel platform a medically relevant range (0.2–100 kPa) can be addressed, which bridges the comparatively elastic hydrogel blends and the rigid scaffolds obtained by hybrid bioprinting.

For successful cell proliferation and tissue development, the choice of the 3D knitted fabric is very important regarding the regulatory effects of the cells’ microenvironment [32,33]. Following the detailed technical investigation, the metabolic activity, cell number, and adipogenic differentiation of MSCs seeded onto warp-knitted PET fabrics were studied. The ability of the cells to proliferate and differentiate within the 3D environment was examined. It is well known that cell responses may differ in 3D microenvironments compared to 2D conditions [34]. For this reason, PET was selected as prototypic material, which was shown to enable cell adhesion [2]. Two days after seeding the MSCs onto the scaffolds, successful cell attachment to the surface of the 3D-PET-scaffold could be observed using 2-photon microscopy (Figure 1A). Using the presto blue assay, an increased metabolic activity of the cells could be detected in the course of the cultivation period, which indicated proliferation. Different volumes were pipetted from a stock suspension of 106 cells/mL, to seed the desired cell numbers on the scaffold (50–400 µL). Presumably, because the swelling volume of the PET structure at 19.6 mm^3^ was not sufficient to absorb the complete media during seeding, with increasing cell numbers, the initial PB conversion of 100,000–400,000 cells were all on similar levels. This suggests that in these approaches the same amount of MSCs adhered to the scaffold. However, we did not measure the PB or CV data on the plates after removal of the scaffold, which could have indicated if the cell suspension traversed the PET scaffold, so that cells just passed through it. Notwithstanding, seeding 50,000 cells in a smaller volume of cell suspension reduced the PB data at Day 0.

It should be critically assessed that PET material, which is not biodegradable, was applied throughout this study. Thus, it cannot be used as a carrier framework for cells in the in vivo model. Further research is therefore required to develop an equivalent biodegradable scaffold, e.g., from PCL or PLA filaments, which can be used for future in vivo studies. Nevertheless, the scaffold and the applied materials were shown to be cytocompatible, exhibited increasing metabolic activity of the cells in the course of the experiment, and indicated high proliferation potential and favorable cell viability. After 14 days of culture, the metabolic activity on the scaffolds initially seeded with 50,000 cells reached the same level as the other groups indicating a similar cell count. This hypothesis was supported by the results of the crystal violet assay 28 days after seeding of the cells that showed similar cell numbers for all groups including the control group of 2D seeded cells. From these results it can be concluded that the PET scaffold had no negative impact on cell proliferation or viability in comparison to a regular 2D culture. The results of the measurements show that there seems to be saturation of the 3D knitted fabric, where an increase in the number of cells cannot be reached after 28 days at the latest. In summary, the construct offered optimal conditions for allowing the cells to proliferate over this period of 28 days.

Previous studies have usually used a high cell count for seeding onto scaffolds. Thus, just a limited statement about the proliferation on tissues was possible [35,36,37,38]. We now showed with different numbers of cells how development of proliferation progresses and a saturation of the 3D knitted fabric is achieved.

In addition, we have succeeded to induce the differentiation of the MSCs into the adipogenic line (Figure 2 and Figure 3). We were able to prove the differentiation by an oil red staining and additionally adipokines analyses by ELISA.

In this work, we were able to identify an optimal three-dimensional framework for proliferation and differentiation of MSCs, which in a further step will be transferred into a biodegradable material.

To sum up, the developed spacer fabric was shown to be a promising platform technology to biofabricate textile-reinforced hydrogel structures with pronounced and tunable mechanical, morphological, as well as biological features. In the future, this platform technology can be applied for a broad range of tissue engineering applications, including load bearing tissues (cartilage or bone) for regenerative medicine as well as soft tissues for reconstructive surgery.

## Figures and Tables

**Table 1:** Detailed information on the warp-knitting process of the textile scaffolds. The table lists the lapping of the cover areas as well as the pile yarn geometry in chain notation.

Scaffold Area	Warp-Knitting Chain Notation	EAC Value
Lower Side	1-0/1-2// 1-2/1-0//	EAC 16
Upper Side	1-0-0-0/1-2-2-2/1-0-0-0/1-2-2-2/3-4-4-4/3-2-2-2/3-4-4-4/3-2-2-2// 3-4-4-4/3-2-2-2/3-4-4-4/3-2-2-2/1-0-0-0/1-2-2-2/1-0-0-0/1-2-2-2//	EAC 16
Pile Yarn I	1-0/2-3//	-
